# Motor Deficits in Schizophrenia Quantified by Nonlinear Analysis of Postural Sway

**DOI:** 10.1371/journal.pone.0041808

**Published:** 2012-08-01

**Authors:** Jerillyn S. Kent, S. Lee Hong, Amanda R. Bolbecker, Mallory J. Klaunig, Jennifer K. Forsyth, Brian F. O’Donnell, William P. Hetrick

**Affiliations:** 1 Department of Psychological and Brain Sciences, Indiana University, Bloomington, Indiana, United States of America; 2 Department of Biomedical Sciences, Ohio University, Athens, Ohio, United States of America; 3 Department of Psychiatry, Indiana University School of Medicine, Indianapolis, Indiana, United States of America; 4 Department of Cognitive Neuroscience, Ludwig Maximilian University of Munich, Munich, Germany; 5 Department of Psychology, University of California Los Angeles, Los Angeles, California, United States of America; 6 Larue D. Carter Memorial Hospital, Indianapolis, Indiana, United States of America; University of Medicine & Dentistry of NJ - New Jersey Medical School, United States of America

## Abstract

Motor dysfunction is a consistently reported but understudied aspect of schizophrenia. Postural sway area was examined in individuals with schizophrenia under four conditions with different amounts of visual and proprioceptive feedback: eyes open or closed and feet together or shoulder width apart. The nonlinear complexity of postural sway was assessed by detrended fluctuation analysis (DFA). The schizophrenia group (n = 27) exhibited greater sway area compared to controls (n = 37). Participants with schizophrenia showed increased sway area following the removal of visual input, while this pattern was absent in controls. Examination of DFA revealed decreased complexity of postural sway and abnormal changes in complexity upon removal of visual input in individuals with schizophrenia. Additionally, less complex postural sway was associated with increased symptom severity in participants with schizophrenia. Given the critical involvement of the cerebellum and related circuits in postural stability and sensorimotor integration, these results are consistent with growing evidence of motor, cerebellar, and sensory integration dysfunction in the disorder, and with theoretical models that implicate cerebellar deficits and more general disconnection of function in schizophrenia.

## Introduction

Motor abnormalities were noted in some of the first descriptions of schizophrenia and incorporated into early characterizations of this complex disorder [1]. Subsequently, substantial empirical evidence of motor deficits in individuals with schizophrenia has accumulated. Recently, interest in the relationship between motor abnormalities, cerebellar function, and the cardinal cognitive and affective symptoms of the disorder has emerged. The present study investigated the nature of cerebellar-dependent motor deficits in individuals with schizophrenia using quantitative assessment of postural sway and a nonlinear dynamical systems analytical approach.

### Motor Abnormalities in the Schizophrenia Spectrum

Schizophrenia is associated with increased involuntary movements [2] and with neurological soft signs (NSS), which are neurological abnormalities in sensory integration, motor coordination, sequencing complex motor acts, and primitive reflexes [3]. Whereas evidence suggests that specific pathways in the basal ganglia (most notably involving the striatum) underlie gross movement abnormalities such as hyperkinesias [4], NSS are by definition not localizable to any single neural substrate [3]. Motor dysfunction is also documented in populations at risk for schizophrenia [5]–[9], and neuromotor dysfunction in individuals with schizophrenia and their relatives is associated with large effect sizes compared to other well-established candidate endophenotypes [10].

### Cerebellar Dysfunction in Schizophrenia

While NSS are traditionally considered nonlocalizing, there is evidence for a specific role of the cerebellum in NSS. For example, there is evidence that cerebellar volume and NSS severity are inversely related [11]. Increased incidence of cerebellum-specific neurologic signs has also been reported in schizophrenia [12]–[13], with Varambally and colleagues classifying 78% of subjects into antipsychotic-naïve schizophrenia or control categories using cerebellar signs [13].

Additionally, there are neuroanatomical and behavioral findings that indicate cerebellar dysfunction in schizophrenia, including structural abnormalities in the cerebellum [14]. Individuals with schizophrenia demonstrate abnormal performance on cerebellar-dependent tasks, including eyeblink conditioning [15]–[16], tone-paced finger tapping [17], and postural sway [18]. Similarly, schizophrenia is associated with abnormalities in behaviors that may also be related to cerebellar function, such as eye movement [19] and gait [20].

Cerebellar impairments in schizophrenia are particularly important when considered in the context of the theory of cognitive dysmetria. According to this theory, the reciprocal connections between the cerebellum and the frontal cortex (mediated by the thalamus) coordinate and sequence motor and cognitive information in a well-timed, fluid manner. This critical circuitry is referred to as the cortico-cerebellar-thalamic-cortical circuit (CCTCC), and there is much evidence of abnormalities in the nodes of the CCTCC in schizophrenia (see [21]–[22] for review). The result is cognitive dysmetria, or a disruption in the fluid temporal coordination of cognition, perception, and motor behavior, and deficits may emerge in any combination of cognitive, perceptual or behavioral domains [21]–[22].

As reviewed above, there are substantial theoretical implications for, as well as compelling empirical evidence of, motor (and specifically cerebellar) abnormalities in schizophrenia. However, certain confounds exist that have historically tempered enthusiasm for these important lines of research. Various authors have acknowledged skepticism of the presence of motor deficits in schizophrenia in light of the documented effects of dopamine-blocking antipsychotics on the motor system [21], [9]. This skepticism persists despite the frequently replicated finding of motor deficits in unmedicated and at-risk populations [23], [2], [5]–[6] and even the observation of motor abnormalities prior to the development of antipsychotic medication [1]. With regard to cerebellar deficits, some authors have suggested that the deterioration of cerebellar tissue that accompanies long-term alcohol dependence [24]–[25] may account for or exacerbate the cerebellar abnormalities documented in schizophrenia [26]–[27]. The current study will investigate cerebellar deficits in schizophrenia while also investigating the effects of these potential confounds.

### Dynamic Analyses and their Benefits

Studies of motor dysfunction in schizophrenia have relied primarily on conventional (normality-based) statistical methods. In addition to studying group differences in the magnitude and variance of motor behavior, the present study also examined differences in the time-dependent *pattern* of motor behavior. This approach expands the breadth of information on motor abnormalities in schizophrenia, and may provide unique insight into the mechanism of the dysfunction.

Nonlinear dynamical systems approaches offer such benefits by illuminating the emergent properties of a system [28]. Physiological processes can be conceptualized as complex dynamical systems, as they are constantly evolving processes comprised of many interacting components and sub-processes. In their healthiest states, natural processes are characterized by a specific type of complexity called self-similarity. A shape or process that is self-similar has the same pattern repeating across all scales of magnitude [28]. For example, blood vessels and neural dendritic arborizations are self-similar structures found in animal anatomy. Self-similarity can also occur across time, where seemingly simple or random processes exhibit statistical patterns (e.g., variance) that repeat across many time scales. Physiological processes such as heart rate and respiration exhibit self-similar dynamics [29]–[30]. A perfectly self-similar process begins to resemble pure randomness as it increases in complexity, and becomes more deterministic (more predictable in pattern) as it loses complexity.

When components of a nonlinear system begin to falter due to disease or dysfunction, both the input of individual components and some of the interactions between them begin to disappear from the system. As a result, the process becomes less complex and a stereotyped pattern emerges due to the fact that there are now fewer components and interactions available to generate the complex output [31]. Crucially, this loss of complexity can occur without a change in mean or standard deviation [28].

There are several novel contributions that will arise from the application of a nonlinear analysis to motor behavior in schizophrenia. Specifically, the investigation of the time-dependent pattern of motor behavior allows for the investigation of dependent variables such as complexity and self-similarity that have yet to be explored in the domain of motor dysfunction in schizophrenia. Given that these variables are emerging as important indicators of dysfunction in the medical field (cf. [28], [31]), it is of great interest to investigate whether measures of complexity and self-similarity will similarly distinguish healthy from pathological systems and processes in the realm of psychiatry. In addition, the application of a nonlinear analysis to postural sway specifically has the potential to yield information about the underlying mechanism of the disturbance in this motor behavior in schizophrenia. Given that nonlinear dynamical systems are comprised of complex interactions between components, the process of postural control is particularly well-suited to this analysis given the complex system formed by the various sensory inputs and their respective sub-components and interactions. By manipulating the input of certain components of the postural control system (i.e. vision or proprioception) and observing the subsequent change in the complexity of postural sway, information regarding the integrity and the unique contribution of these individual inputs can be revealed.

### The Present Study

The present study expands on the important but understudied area of motor dysfunction in schizophrenia through the application of a nonlinear analysis to postural sway. Postural sway is the area over which one’s center of pressure moves during the process of maintaining upright posture. Unlike the more gross assessments of upright balance found in NSS batteries, very sensitive instruments are required to quantitatively measure the small-scale alterations in center of pressure characteristic of postural sway, which occurs on a scale of millimeters. This fine motor behavior requires cerebellar integration of visual, proprioceptive, and vestibular information to correctly orient the body in space and maintain an upright standing position [32], and integrity of the cerebellum is essential to maintaining proper postural control. Given the importance of the cerebellum in both postural control and the CCTCC, assessment of postural sway has excellent potential to be a useful tool in studying the integrity of this important node in schizophrenia.

In the only such study to date, Marvel and colleagues [18] reported that sway area was increased in individuals with schizophrenia compared to controls. Building on this work, the present study examined sway area using the conventional approach to analysis but also applied nonlinear methods. Specifically, center-of-pressure (COP) data were analyzed with detrended fluctuation analysis (DFA) [33] to index the degree of complexity and self-similarity in postural sway. In addition to quantifying the overall complexity of postural sway, DFA was also used to explore the contribution of visual and proprioceptive information through examining changes in postural sway complexity in response to manipulations of these inputs, both of which have characteristic dynamics and hypothesized effects on sway complexity in response to manipulation. Through exploring the contribution of these specific sensory processes to motor dysfunction in schizophrenia, information about the mechanism of motor disturbances in this population can be revealed.

The present study also investigated the relationship of postural sway abnormalities to symptom severity. In a review of NSS in schizophrenia, Bombin and colleagues [3] concluded that shared variance would be expected between NSS and clinical symptoms if overlapping structural and/or functional brain abnormalities instantiate these phenomena. In addition, the authors stated that such a relationship would make the case that NSS be considered an “essential feature” of schizophrenia [3]. Ultimately, the authors concluded that *negative* symptoms are most consistently associated with NSS in schizophrenia. The current study investigated whether this relationship between motor abnormalities and symptom severity would be present in the domain of postural control by assessing the strength of the correlations between postural sway dependent variables and subscales of the Positive and Negative Syndrome Scale (PANSS) [34].

Given the empirical evidence of cerebellar deficits in schizophrenia as well as the theoretical importance of the cerebellum as a node in the CCTCC, it was hypothesized that abnormal postural sway would be present in individuals with schizophrenia, manifested both as increased postural sway (as previously reported [18]), and as decreased complexity [28]. In addition, it was hypothesized that individuals with schizophrenia would demonstrate abnormal sensory integration, as evidenced by abnormal changes in postural sway complexity in response to manipulations of visual and proprioceptive inputs. It was also hypothesized that these group differences would not be driven by the effects of antipsychotic medication or past alcohol dependence. Finally, in light of previous findings of negative symptom correlates of motor abnormalities in schizophrenia, it was hypothesized that the severity of postural sway abnormalities would be associated with the severity of negative symptoms.

## Methods

### Participants

Twenty-seven individuals (16 male) with schizophrenia (n = 14) or schizoaffective disorder (n = 13) comprised the schizophrenia sample and were compared with 37 non-psychiatric comparison subjects (13 male) (Table 1). All participants provided written informed consent and were aware that declining to participate would not negatively impact their treatment or result in any other disadvantages. This study was approved by the Indiana University IRB and was conducted in accordance with the Declaration of Helsinki. We assessed the level of psychotic symptoms to identify individuals who were not oriented to person, place, and time, and, by this standard, might not be capable of providing informed consent. Furthermore, in no case was an individual who has been found incompetent to make decisions on their own behalf, as determined by a court of law, enrolled into this study. We emphasized to each participant that the study could be terminated at any time at the participant's request without jeopardizing benefits to which they would otherwise be entitled. Specifically, participants were informed that they would be reimbursed for the total amount of time contributed to the study even if they chose to discontinue their participation before data collection was completed.

**Table 1:** Summary statistics for all diagnostic groups.

Characteristics	Control	Schizophrenia
Age (years), Mean (SD)	40.2 (11.4)	41.9 (9.1)
Height (inches), Mean (SD)	67.0 (4.5)	67.6 (4.3)
Weight (pounds), Mean (SD)	194.3 (53.7)	196.3 (40.2)
BMI, Mean (SD)	30.7 (8.8)	30.3 (5.9)

Diagnoses were determined through the Structured Clinical Interview for DSM-IV Axis I disorders (SCID-I) [35] and review of health records. The PANSS [34] was administered to 25 of the participants with schizophrenia (Table 2) and all participants with schizophrenia were administered the Abnormal Involuntary Movement Scale (AIMS) [36] and none scored positive (defined by the AIMS as mild abnormality in two movements or moderate or severe abnormality in one movement). Trained psychodiagnosticians administered all clinical symptom and diagnostic scales, and kappa inter-rater reliability has been 0.95 for the distinction between schizophrenia, mood disorders, or other disorders in this laboratory.

**Table 2:** Summary of PANSS subscale scores.

PANSS subscale	Average score
Positive, Mean (SD)	17.1 (6.3)
Negative, Mean (SD)	11.7 (4.0)
General, Mean (SD)	30.2 (7.7)
Total, Mean (SD)	58.9 (15.3)

Exclusion criteria included current drug or alcohol abuse or dependence, and report of loss of consciousness. Control participants were excluded for past drug or alcohol dependence. A one-way ANOVA revealed no significant interactions between diagnosis (schizophrenia versus control) and age, height, weight or body mass index (BMI), and Pearson’s chi-square test revealed no systematic relationship between gender and diagnosis. Medication regimens in the clinical sample were as follows: atypical antipsychotics (n = 6); typical antipsychotics (n = 6); mood stabilizers or elevators (n = 14); and unmedicated (n = 6).

### Procedure

Participants were asked to stand as still as possible with their arms resting comfortably at their sides on an AMTI Accusway (Watertown, MA) force platform under four conditions with different vision (eyes open vs. closed) and base (feet together vs. shoulder-width apart) conditions. Two minute recordings of center of pressure (COP) were made for each of the following conditions for each participant in this order: eyes open, closed base (EOCB); eyes closed, closed base (ECCB); eyes closed, open base (ECOB); and eyes open, open base (EOOB), sampled at 50 Hz along the anteroposterior and mediolateral axes.

### Data Analysis

**Figure 1:**
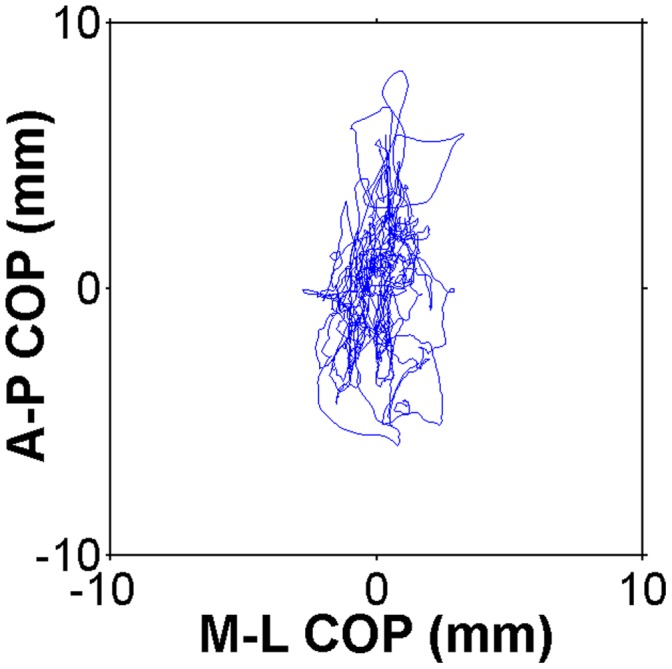
Example sway area plot. COP movement in the mediolateral direction is shown on the X axis, and COP movement in the anteroposterior direction is shown on the Y axis.

A 9^th^ order Butterworth lowpass filter with a 25 Hz cutoff frequency was applied to recordings to isolate the low-frequency postural sway process. COP and its 95% confidence area encompassed by COP movement was measured using principal component analysis (PCA) [37] (Figure 1). The use of an ellipse encompassing 95% of all data points along the X and Y axes is a well-accepted measure of the magnitude of postural sway [38]–[39]. To determine whether group differences in the magnitude of postural sway were driven primarily by one direction, the standard deviation of movement away from the average COP was analyzed for each axis.

The COP data were also subjected to detrended fluctuation analysis (DFA) to quantify the complexity of the postural sway time series. DFA was applied to the mediolateral and anteroposterior dimensions independently. These dimensions are known to be driven by different muscles and joint action and are differently affected by the loss of sensory inputs. Due to these fundamental differences in input and output, movement along the mediolateral and anteroposterior dimensions possesses distinct characteristic dynamics [40]. Information about the dynamics of postural sway is therefore maximized when DFA is applied separately to these axes of motion.

**Figure 2:**
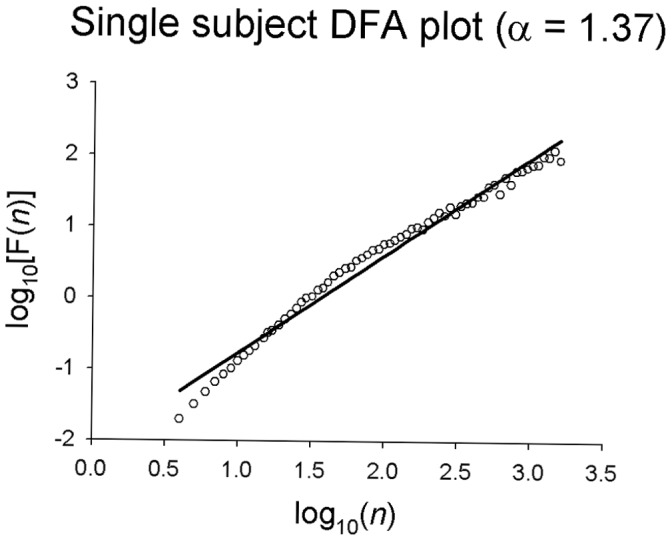
Single subject DFA plot. The log of window size is shown on the X axis and the log of variance as a function of window size is shown on the Y axis.

DFA indexes the relative distribution of variance within the data across a range of different time scales. This produces a profile of the time series in terms of the correlation/anticorrelation properties of the signal, quantified by the rate of growth in fluctuation of variance as a function of increasing time scales. The growth in fluctuation magnitude across time scales is indexed by the slope of this function in double-logarithmic space (known as the á-value) (Figure 2). á-values greater than 0.5 indicate that a time series is characterized by autocorrelation on some time scale, while signals that are anticorrelated have á-values less than 0.5. An á-value of 1 is present in 1/f noise and characterizes fractals and healthy physiological systems, indicating the maximum degree of self-similarity in a signal. This is a unique pattern of complexity, as the magnitude of the fluctuations grows in direct proportion to the time-scale on which the fluctuations are measured. Decreased complexity in the system results in higher á-values; for example, the á-value describing Brownian noise is 1.5. In this case, a greater proportion of the fluctuations within the time series occur at longer time scales in comparison to shorter ones and the output of the system is more deterministic than a self-similar signal. á-values decrease as a system becomes increasingly random, with a completely random signal classified as white noise with an á-value of 0.5 (indicating that the magnitude of the fluctuations is virtually similar at all time scales).

A major advantage of applying DFA is the sensitivity of this analysis to the differential contributions of sensory inputs that occur on different timescales. The current study manipulates both visual and proprioceptive input. Visual information is relatively slow sensory input, occurring at lower frequencies and on slower time scales [41]. The removal of low-frequency visual input should increase complexity of postural sway as quantified by DFA because faster, shorter-frequency inputs will increase in contribution to postural control, therefore rendering a more complex postural sway signal output. Proprioception occurs on faster timescales and is a high-frequency input to the postural control system [42]. When proprioceptive information is increased (as in the open base condition), postural sway complexity should increase as a result of more high-frequency proprioceptive input contributing to postural control. By applying DFA to postural sway data collected under different vision and proprioception conditions and examining how these manipulations affect postural sway complexity, the underlying sensory processes contributing to motor function and dysfunction can be elucidated.

### Statistical Analysis

Repeated measures ANOVAs were performed on sway area and DFA in the mediolateral (ML) and anteroposterior (AP) directions. Eyes (open or closed) and base (open or closed) were within-subjects factors and group was the between subjects factor.

To explore the potential confounding effects of alcohol on postural sway in schizophrenia, all ANOVAs were repeated excluding schizophrenia participants dually diagnosed for past alcohol dependence (n = 14). All ANOVAs also were repeated comparing singly- (n = 13) and dually-diagnosed (n = 14) individuals with schizophrenia. To address the potential effect of dopamine-blocking antipsychotic medication, chlorpromazine equivalent dosages were computed for all participants taking medications for which accepted chlorpromazine conversions had been established (n = 17) [43]–[44]. Relationships between antipsychotic medication dosage and postural sway dependent variables were examined with Pearson product-moment correlations. All ANOVAs were repeated comparing clinical participants currently taking antipsychotics (n = 21) and those currently not (n = 6). Finally, Pearson product-moment correlations were computed to assess relationships between symptoms (measured by the PANSS [34]) and sway dependent variables. Where Pearson product-moment correlations are reported, data were screened for outliers, defined as 3 SDs above the mean, and appropriate adjustments to degrees of freedom are shown in the results.

## Results

### Sway Area

Analysis of sway area revealed a main effect of group, with mean sway area larger in schizophrenia participants (*M* = 75.44 mm^2^, *SE* = 11.43 mm^2^) compared to controls (*M* = 31.83 mm^2^, *SE* = 9.76 mm^2^) (F(1,62) = 8.42, p = 0.005, η_p_^2^ = 0.12), indicating abnormalities in postural control. There were main effects of eyes (F(1,62) = 13.47, p = 0.001, η_p_^2^ = 0.18), and base (F(1,62) = 5.27, p = 0.025, η_p_^2^ = 0.08), indicating that sway area was greater when eyes were closed (eyes open: *M* = 35.95 mm^2^, *SE* = 4.17 mm^2^; eyes closed: *M* = 71.32 mm^2^, *SE* = 11.92 mm^2^) and when feet were adjacent in the closed base condition (open base: *M* = 43.21 mm^2^, *SE* = 9.29 mm^2^; closed base: *M* = 64.05 mm^2^, *SE* = 8.24 mm^2^), respectively.

**Figure 3:**
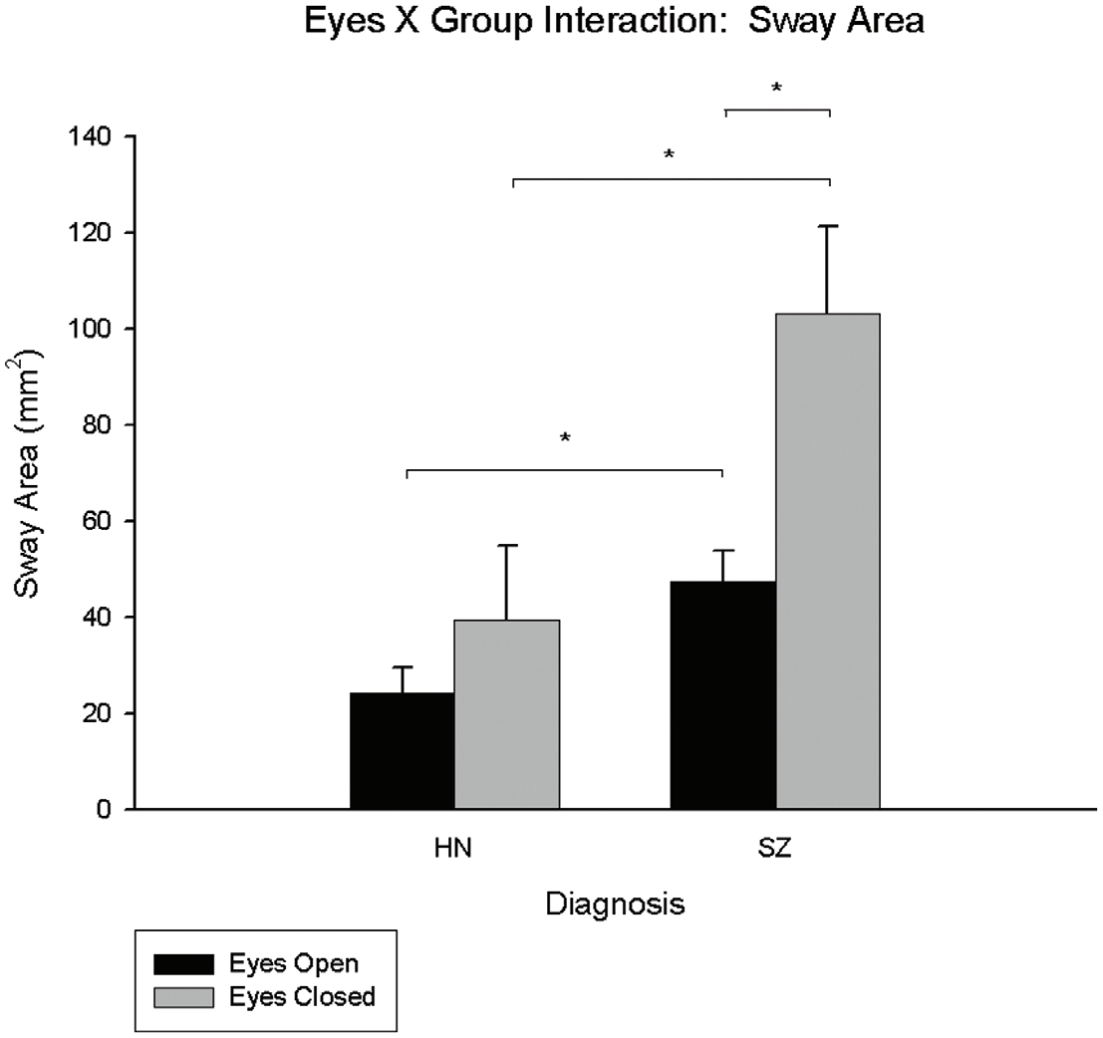
Eyes X group interaction for healthy non-psychiatric controls and schizophrenia participants for sway area. The HN bars represent the data for the healthy non-psychiatric controls and the SZ bars represent the data for the participants with schizophrenia; significant pair-wise comparisons are starred.

There was a significant eyes X group interaction (F(1,62) = 4.431, p = 0.039, η_p_^2^ = 0.07) (Figure 3). Post-hoc pair-wise comparisons indicated that the schizophrenia group had significantly larger sway area in the eyes closed compared to open condition, but this pattern was absent in controls (see Supporting Information S1). These results indicate that the postural sway of schizophrenia participants was more affected by the loss of visual information than that of controls. When sway area was separated into movement across the mediolateral (ML) and anteroposterior (AP) axes using the standard deviation of motion on each axis away from average COP, the significant main effect of diagnosis remained for each axis. The eyes X diagnosis interactions dropped to trend level for both axes (ML: p = 0.085; AP: p = 0.064).

### Detrended Fluctuation Analysis: Mediolateral Direction

Analysis of DFA-ML values revealed a significant main effect of eyes (F(1,62) = 5.79, p = 0.010, η_p_^2^ = 0.085) and base (F(1,62) = 78.725, p<0.001, η_p_^2^ = 0.559). As expected [41], average DFA values were smaller (and postural sway was therefore more complex) when eyes were closed (*M = *1.371, *SE = *0.013) compared to open (*M = *1.388, *SE = *0.012). When vision, a slower sensory input that provides lower-frequency information on longer timescales is removed, faster, higher frequency sensory processes such as proprioception dominate the control of posture and therefore generate a more complex output. Conversely, average DFA values were larger (and postural sway was less complex) in the closed (*M = *1.436, *SE = *0.011) compared to the open base (*M = *1.323, *SE = *0.016) conditions. This is also consistent with expectations, as proprioceptive information is transmitted at a very high frequency [42], so postural sway was less complex with decreased proprioceptive feedback given that fewer postural corrections were made on shorter time scales when participants stood with their feet together. The main effect of group approached significance (F(1,62) = 2.919, p = 0.093, η_p_^2^ = 0.045), with the schizophrenia group exhibiting less complex postural sway.

### Detrended Fluctuation Analysis: Anteroposterior Direction

Analysis of DFA-AP values revealed a main effect of eyes (F(1,62) = 18.33, p<0.001, η_p_^2^ = 0.23), a main effect of base (F(1,62) = 5.00, p = 0.029, η_p_^2^ = 0.08), and a significant eyes X base interaction (F(1,62) = 47.23, p<0.001, η_p_^2^ = 0.43) (see Supporting Information S2). There was no main effect of group and there were no group interactions.

### Analysis of Past Alcohol Dependence

When individuals with schizophrenia who also had a history of alcohol dependence were excluded, results were unchanged for the analysis of sway area.

Main effects of eyes and base of the same direction remained significant for the analysis of DFA-ML values. However, a significant main effect of group emerged (F(1,48) = 4.151, p = 0.047, η_p_^2^ = 0.080), with schizophrenia participants without a history of alcohol dependence exhibiting higher DFA-ML values (*M* = 1.42, *SE* = 0.027) compared to controls (*M* = 1.36, *SE* = 0.016), and therefore less complex postural sway along this axis. This decrease in postural sway complexity in the schizophrenia group indicates that there are potentially fewer and/or slower sensory components contributing to the process of postural control.

For DFA-AP values, the main effect of eyes dropped to marginal significance (F(1,48) = 3.924, p = 0.053, η_p_^2^ = 0.076), while the main effect of base and eyes X base interaction remained significant (see Supporting Information S3).

**Figure 4:**
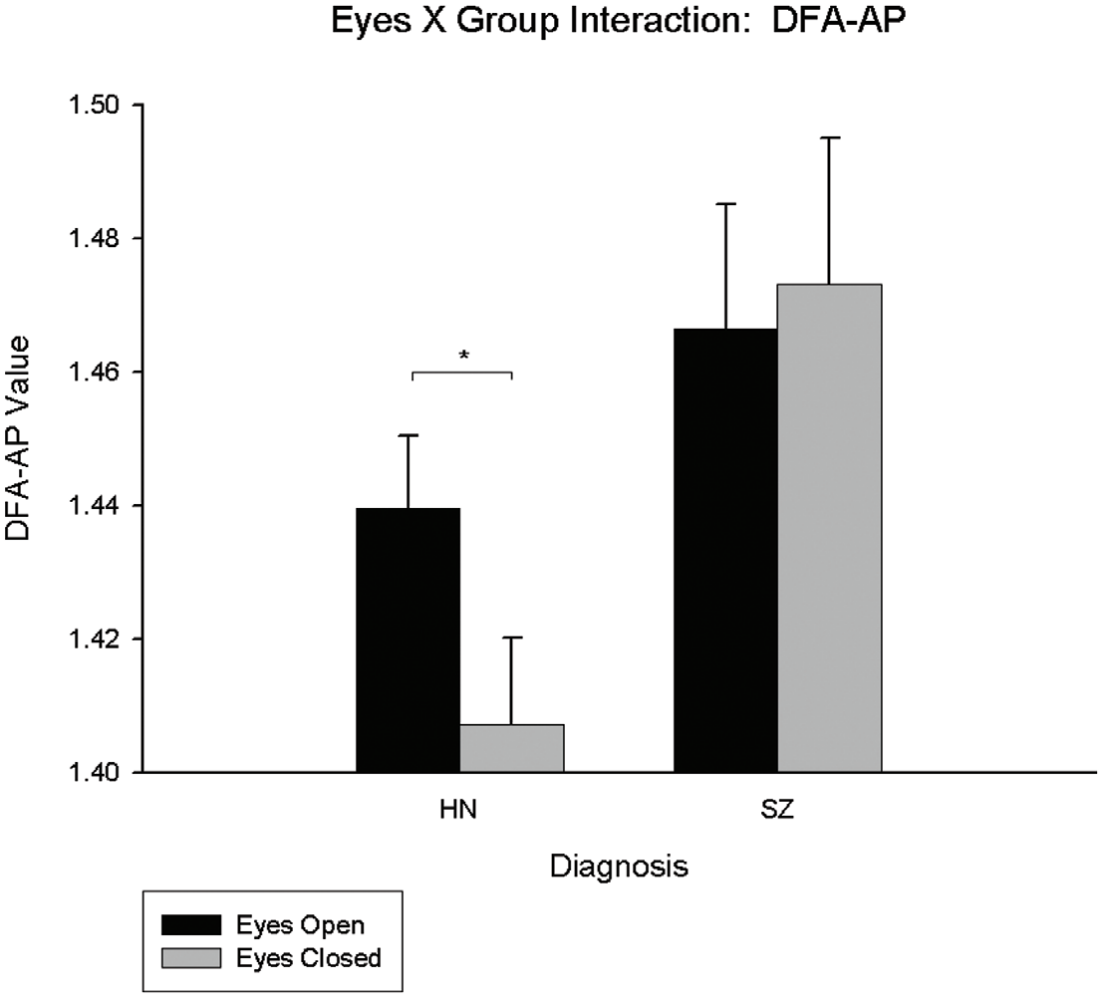
Eyes X group interaction for non-psychiatric controls and singly diagnosed schizophrenia participants for DFA-AP. The HN bars represent the data for the healthy non-psychiatric controls and the SZ bars represent the data for singly-diagnosed participants with schizophrenia; significant pair-wise comparisons are starred.

A significant DFA-AP main effect of group emerged (F(1,48) = 4.204, p = 0.046, η_p_^2^ = 0.081), indicating decreased complexity of postural sway in singly-diagnosed individuals with schizophrenia (*M* = 1.47, *SE* = 0.019) compared to controls (*M* = 1.42, *SE* = 0.012). There was also a significant eyes X group interaction (F(1,48) = 8.888, p = 0.004, η_p_^2^ = 0.156), indicating that only controls demonstrated the expected increase in complexity upon the removal of visual input (Figure 4) (see Supporting Information S4). When all analyses were repeated comparing singly and dually diagnosed schizophrenia participants, no main effects of group or group interactions emerged for sway area or DFA-ML. For DFA-AP values, there was a significant eyes X group interaction (F(1,25) = 5.675, p = 0.025, η_p_^2^ = 0.185), in which only dually diagnosed individuals demonstrated the expected increase in complexity when eyes were closed compared to open (see Supporting Information S5).

### Analysis of Medication Effects

There were no significant correlations between chlorpromazine equivalent values and any condition for sway area, DFA-ML, or DFA-AP. Repeating all analyses comparing participants with schizophrenia on (n = 21) and off (n = 6) antipsychotic medication revealed no main effects of group or group interactions for any postural sway dependent variable.

### Symptom Severity and Postural Sway

**Figure 5:**
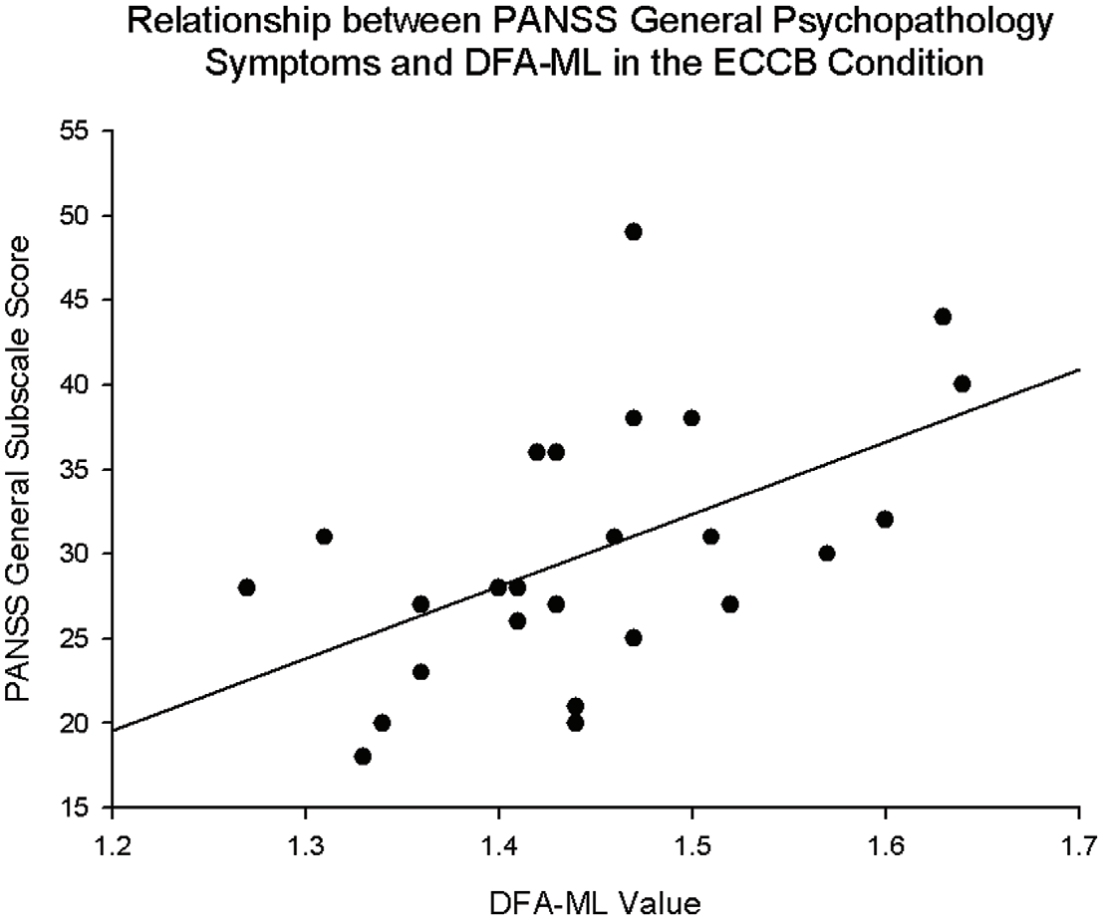
Correlation between PANSS general psychopathology subscale scores and DFA-ML values in the ECCB condition. r(23) = 0.531, p = 0.006.

There was a significant correlation between sway area in the EOOB condition and the general psychopathology (r(22) = 0.466, p = 0.022) PANSS factor. However, this correlation did not survive á-level Bonferroni correction for the 4 sway conditions (p>0.0125). There were also significant correlations between DFA-ML values in the ECCB condition and the negative (r(23) = 0.404, p = 0.045) and general psychopathology (r(23) = 0.531, p = 0.006) PANSS factors, with the correlation between DFA-ML values and the general psychopathology PANSS factor surviving Bonferroni correction (p<0.0125) (Figure 5).

## Discussion

The application of traditional and nonlinear analyses to postural sway indicated that participants with schizophrenia exhibited increased but less complex postural sway compared to controls. Schizophrenia was associated with inefficient adaptation to a loss of visual information (i.e., eye closure) during maintenance of posture. These abnormalities are likely due to abnormal integration of sensory information from a variety of modalities critical to the process of postural control (i.e., vision, proprioception, and the vestibular sense). Importantly, the findings could not be explained by medication effects or a history of alcohol dependence in the schizophrenia sample. Finally, postural sway deficits were associated with increased symptom severity. Given the importance of the cerebellum in postural control, the observed sway abnormalities in schizophrenia are consistent with evidence of cerebellar and sensory integration dysfunction in schizophrenia.

### Sway Area

Analyses of sway area replicated previous findings [18] showing an overall increase in sway area in individuals with schizophrenia compared to controls. However, Marvel and colleagues [18] reported a base by group interaction for sway area, whereas the present results indicated an eyes by group interaction. There is significant heterogeneity in schizophrenia samples, which may account for these differences. The present results are consistent with a study of postural sway in bipolar disorder [45], wherein an eyes by group interaction was similarly observed for sway area and cerebellar deficits have also been reported [46]. The replication of postural disturbances in schizophrenia in the form of increased sway area suggests that dysfunctional postural control is indeed part of the constellation of motor abnormalities present in schizophrenia.

### Detrended Fluctuation Analysis

DFA provides a more complete characterization of postural sway than sway area alone because it reveals information about the *structure* of the postural sway process. Specifically, DFA indexes the complexity of postural sway and can be informative as to how many and what type of component processes underlie the maintenance of posture. The finding of decreased postural sway complexity in schizophrenia in both the mediolateral and anteroposterior directions is consistent with findings of decreased complexity in a variety of pathological physiological processes [28], [31]. Decreased complexity indicates that high frequency components are missing from the postural control process and may indicate improper integration of visual, proprioceptive, and vestibular sensory information critical for postural stability. Interestingly, the group effect of decreased complexity reached significance when dually diagnosed schizophrenia subjects were excluded. This means that past alcohol dependence was not driving any observed effects and, in fact, may have contributed to increased noise in the DFA values due to the potentially heterogeneous effects of alcohol-related degradation of relevant neuronal substrates. This hypothesis is consistent with the larger standard deviations in average DFA values in the dually diagnosed compared to singly diagnosed schizophrenia sample.

In addition, an eyes by group interaction emerged for DFA-AP values when dually diagnosed schizophrenia participants were excluded. This interaction was driven by greater complexity in the eyes closed compared to the eyes open condition in controls, but not in schizophrenia participants. Removal of visual input should result in increased sway complexity because visual input normally provides low-frequency feedback for postural sway on longer timescales [41]. The removal of visual input should result in increased reliance on higher-frequency feedback occurring on shorter time scales (for example, proprioception [42]), thereby resulting in a postural sway signal characterized by more complexity. The observed interaction could have resulted from two candidate abnormalities in the schizophrenia sample: (1) a decreased range of time scales available on which corrections to postural sway can be made; and/or (2) an inability to properly integrate higher-frequency feedback such as proprioception (cf. Bolbecker and colleagues [45]).

### Effects of Past Alcohol Dependence

Results from the analysis of sway area were unchanged when schizophrenia participants with a history of alcohol dependence were excluded. However, additional group effects emerged for DFA upon exclusion of these individuals. The only difference that emerged when singly and dually diagnosed schizophrenia participants were compared on all sway dependent variables was an eyes by group interaction for DFA-AP, in which the dually diagnosed group demonstrated the predicted change in complexity between the eyes conditions. These results indicate that the current findings were not driven by the effects of past alcohol dependence-related cerebellar damage in the schizophrenia sample.

### Medication Effects

No results appear to be driven by the effects of antipsychotics on the motor system, as there were no significant correlations between chlorpromazine equivalent dosage values and any of the postural sway dependent variables. Furthermore, there were no group effects or interactions for any of the postural sway dependent variables when the individuals with schizophrenia taking antipsychotic medications were compared to those who were not.

### Clinical Correlates

The general psychopathology subscale of the PANSS correlated with mediolateral DFA in the most difficult condition (ECCB). This finding of symptom correlates of postural sway is consistent with the existing literature [3] in that there is a relationship between motor abnormalities and symptom severity, although the present results indicate a relationship between general psychopathology symptoms rather than negative symptoms (the most consistently reported category of symptoms related to motor dysfunction). While further research on this issue is necessary, the present result suggests a relationship between general psychopathology symptom severity and postural sway deficits in schizophrenia, therefore implying that some portion of the neural circuitry underlying symptoms of schizophrenia and postural sway abnormalities may be shared (cf. Bombin and colleagues [3]).

### Integration with Existing Models

Abnormalities in postural sway in schizophrenia manifested through both larger and less complex postural sway are likely (if not exclusively) indicative of cerebellar dysfunction in schizophrenia. Such dysfunction is consistent with the cognitive dysmetria theory of schizophrenia, in which the cerebellum acts as a general coordinative organ in the CCTCC for both motor and cognitive functions [21]–[22], and the present findings add additional support to the cerebellar dysfunction aspect of this theory of schizophrenia. The findings of abnormal postural adaptation in response to decreased visual input in schizophrenia identifies a more specific sensory integration candidate mechanism of the more general postural sway abnormalities observed in the present study. Such results are consistent with greater theoretical models of schizophrenia as a disconnection syndrome in which abnormalities in integrative circuits underlying cognition and sensorimotor integration contribute to the disturbances in behavior, higher-order cognitive function, and consciousness [47]–[48].

### Limitations and Future Directions

Several limitations of the current study must be addressed. First, although a concerted effort was made to rule out potential confounds of medication regimens, possible effects of the dopamine-blocking properties of antipsychotics on the motor system could not be completely eliminated. While the AIMS is sensitive to general movement abnormalities, other measures such as the Simpson-Angus Extrapyramidal Side Effects Scale [49] and the Barnes Akathisia Rating Scale [50] that are more specifically sensitive to extrapyramidal symptoms should be included in future research to more stringently control for the potential effects of atypical antipsychotics. However, consistent findings of motor abnormalities in medication-naïve and at-risk populations (such as relatives and individuals with schizotypal personality disorder) [2], [5]–[9], [23] and the present study’s thorough exploration of the relationship between antipsychotic dosage and postural sway dependent variables and the comparison between medicated and unmedicated participants suggests that the current results are not being driven by medication effects. In addition, the observed correlation between postural sway and symptom severity must be interpreted with caution, as neuroleptic medication could have influenced both variables.

Second, because there is strong evidence for the involvement of the cerebellum in integrating visual, proprioceptive, and vestibular information for the maintenance of posture [32] as well as for cerebellar deficits in schizophrenia [14]–[17], it seems reasonable to infer that cerebellar deficits in individuals with schizophrenia could account for the reported results. Even so, other neural structures are involved in postural control and sensory integration, and could contribute to the observed group differences. For example, the basal ganglia are an alternative candidate [51] and are also abnormal in schizophrenia [4]. Therefore, given that the origin of motor abnormalities in schizophrenia (including postural sway deficits) is likely to be multiply determined, future research should aim to elucidate the concurrent contribution of multiple neural regions (i.e., using MRI).

Third, there is evidence to suggest that individuals with schizophrenia age more rapidly than non-psychiatric controls, particularly later in life [52]. Subsequent studies should include a cross-sectional component including different age cohorts to investigate whether aging exacerbates postural sway dysfunction in schizophrenia.

Finally, the results of the current study are consistent with models of schizophrenia that point to cerebellar dysfunction as well as theories postulating functional and structural disconnection. As a result, future studies of motor abnormalities in schizophrenia should aim to examine more integrative functions rather than sensory or motor functions alone.

## Supporting Information

Supporting Information S1 **Sway area eyes X group interaction post-hoc pair-wise comparisons.**
(DOC)

Supporting Information S2 **DFA-AP eyes X base interaction post-hoc pair-wise comparisons.**
(DOC)

Supporting Information S3 **DFA-AP eyes X base interaction (exclusion for past alcohol dependence) post-hoc pair-wise comparisons.**
(DOC)

Supporting Information S4 **DFA-AP eyes X group interaction (exclusion for past alcohol dependence) post-hoc pair-wise comparisons.**
(DOC)

Supporting Information S5 **DFA-AP eyes X group interaction (singly versus dually diagnosed schizophrenia participants) post-hoc pair-wise comparisons.**
(DOC)
