# Supplementary material for: Motor Deficits in Schizophrenia Quantified by Nonlinear Analysis of Postural Sway
Source: PLoS One. 2012 Aug 1;7(8):e41808. doi: 10.1371/journal.pone.0041808 (PMC3411581; doi:10.1371/journal.pone.0041808)
Supplement: Supporting Information S1 — Sway area eyes X group interaction post-hoc pair-wise comparisons. (DOC) [file pone.0041808.s001.doc]

**Supporting Information S1**

Motor Deficits in Schizophrenia Quantified by Nonlinear Analysis of Postural Sway

Jerillyn S. Kent,^1^ S. Lee Hong,^2^ Amanda R. Bolbecker,^1,3^ Mallory J. Klaunig,^4^ Jennifer K. Forsyth,^5^ Brian F. O’Donnell,^1,3,6^ & William P. Hetrick*^1,3,6^

1. Department of Psychological and Brain Sciences, Indiana University, Bloomington, Indiana, United States of America
2. Department of Biomedical Sciences, Ohio University, Athens, Ohio, United States of America
3. Department of Psychiatry, Indiana University School of Medicine, Indianapolis, Indiana, United States of America
4. Department of Cognitive Neuroscience, Ludwig Maximilian University of Munich, Munich, Germany
5. Department of Psychology, University of California Los Angeles, Los Angeles, California, United States of America
6. Larue D. Carter Memorial Hospital, Indianapolis, Indiana, United States of America

*corresponding author: whetrick@indiana.edu (email); 1-812-855-2620 (phone); 1-812-855-2012 (fax)

*Supporting Information S1: Sway area eyes X group interaction post-hoc pair-wise comparisons*

Four post-hoc pair-wise comparisons (α-level adjusted to p < 0.0125) were conducted to compare average sway area in the eyes open compared to closed condition within each group and to compare performance between groups for these two visual conditions. Schizophrenia subjects had significantly larger sway area than controls in both the eyes open (schizophrenia: *M* = 47.6 mm^2^, *SE =* 6.3 mm^2^; control: *M* = 24.3 mm^2^, *SE =* 5.4 mm^2^; Mean Difference = 23.3 mm^2^, *SE =* 8.3 mm^2^, p = 0.007) and eyes closed (schizophrenia: *M*  = 103.3 mm^2^, *SE =* 18.1 mm^2^; control: *M*  = 39.4 mm^2^, *SE = 1*5.5 mm^2^; Mean Difference = 63.9 mm^2^, *SE =* 23.8 mm^2^, p = 0.009) conditions. However, only the participants with schizophrenia had significantly increased sway area in the eyes closed (*M* = 103.3 mm^2^, *SE =* 18.1 mm^2^) compared to open (*M* = 47.6 mm^2^, *SE =* 6.3 mm^2^) conditions (Mean Difference = 55.6 mm^2^, *SE =* 14.7 mm^2^, p < 0.001).
