# Supplementary material for: Motor Deficits in Schizophrenia Quantified by Nonlinear Analysis of Postural Sway
Source: PLoS One. 2012 Aug 1;7(8):e41808. doi: 10.1371/journal.pone.0041808 (PMC3411581; doi:10.1371/journal.pone.0041808)
Supplement: Supporting Information S2 — DFA-AP eyes X base interaction post-hoc pair-wise comparisons. (DOC) [file pone.0041808.s002.doc]

**Supporting Information S2**

Motor Deficits in Schizophrenia Quantified by Nonlinear Analysis of Postural Sway

Jerillyn S. Kent,^1^ S. Lee Hong,^2^ Amanda R. Bolbecker,^1,3^ Mallory J. Klaunig,^4^ Jennifer K. Forsyth,^5^ Brian F. O’Donnell,^1,3,6^ & William P. Hetrick*^1,3,6^

1. Department of Psychological and Brain Sciences, Indiana University, Bloomington, Indiana, United States of America
2. Department of Biomedical Sciences, Ohio University, Athens, Ohio, United States of America
3. Department of Psychiatry, Indiana University School of Medicine, Indianapolis, Indiana, United States of America
4. Department of Cognitive Neuroscience, Ludwig Maximilian University of Munich, Munich, Germany
5. Department of Psychology, University of California Los Angeles, Los Angeles, California, United States of America
6. Larue D. Carter Memorial Hospital, Indianapolis, Indiana, United States of America

*corresponding author: whetrick@indiana.edu (email); 1-812-855-2620 (phone); 1-812-855-2012 (fax)

*Supporting Information S2: DFA-AP eyes X base interaction post-hoc pair-wise comparisons*

Four post-hoc pair-wise comparisons were conducted investigating the differences in DFA-AP values between all four conditions (adjusting the α-value to p < 0.0125). Post-hoc tests revealed that participants had smaller DFA-AP values (more complex sway) when eyes were closed (*M =* 1.399, *SE =* 0.012) compared to open (*M =* 1.454, *SE =* 0.009) when participants were in the closed base condition (Mean Difference = -0.055, *SE* = 0.007, p < 0.001). This pattern of increased complexity when visual input is removed is similar to that exhibited in the DFA-ML values. The absence of this pattern in the open base condition could be due to a ceiling effect of open base in the AP direction; i.e., the presence or absence of visual input does not appreciably change the complexity of sway when feet are shoulder-width apart. In the eyes open condition, DFA-AP values were smaller (postural sway was more complex) in the open base (*M =* 1.434, *SE =* 0.011) compared to the closed base (*M =* 1.454, *SE =* 0.009) condition (Mean Difference = -0.020, *SE* = 0.007, p = 0.006). This pattern of increased complexity with increased proprioceptive information is congruent with DFA-ML findings. However, in the eyes closed condition, DFA-AP values were larger (i.e., postural sway was less complex) in the open base (*M =* 1.442, *SE =* 0.013) compared to the closed base (*M =* 1.399, *SE =* 0.012) condition (Mean Difference = 0.043, *SE* = 0.007, p < 0.001). It is unclear why postural sway would be more complex in the closed base compared to the open base condition while eyes are closed. This difference in the relationship between proprioceptive information and postural sway complexity between the eyes open and closed conditions is the source of the eyes X base interaction for DFA-AP.
