# Supplementary material for: Motor Deficits in Schizophrenia Quantified by Nonlinear Analysis of Postural Sway
Source: PLoS One. 2012 Aug 1;7(8):e41808. doi: 10.1371/journal.pone.0041808 (PMC3411581; doi:10.1371/journal.pone.0041808)
Supplement: Supporting Information S4 — DFA-AP eyes X group interaction (exclusion for past alcohol dependence) post-hoc pair-wise comparisons. (DOC) [file pone.0041808.s004.doc]

**Supporting Information S4**

Motor Deficits in Schizophrenia Quantified by Nonlinear Analysis of Postural Sway

Jerillyn S. Kent,^1^ S. Lee Hong,^2^ Amanda R. Bolbecker,^1,3^ Mallory J. Klaunig,^4^ Jennifer K. Forsyth,^5^ Brian F. O’Donnell,^1,3,6^ & William P. Hetrick*^1,3,6^

1. Department of Psychological and Brain Sciences, Indiana University, Bloomington, Indiana, United States of America
2. Department of Biomedical Sciences, Ohio University, Athens, Ohio, United States of America
3. Department of Psychiatry, Indiana University School of Medicine, Indianapolis, Indiana, United States of America
4. Department of Cognitive Neuroscience, Ludwig Maximilian University of Munich, Munich, Germany
5. Department of Psychology, University of California Los Angeles, Los Angeles, California, United States of America
6. Larue D. Carter Memorial Hospital, Indianapolis, Indiana, United States of America

*corresponding author: whetrick@indiana.edu (email); 1-812-855-2620 (phone); 1-812-855-2012 (fax)

*Supporting Information S4: DFA-AP eyes X group interaction (exclusion for past alcohol dependence) post-hoc pair-wise comparisons*

Four post-hoc pair-wise comparisons were conducted in which the eyes open versus closed conditions were examined both within and between groups (α-level adjusted to p < 0.0125). As expected [41], in controls there were significantly larger DFA-AP values (less complexity) in the eyes open (*M =* 1.440, *SE =* 0.011) compared to eyes closed (*M =* 1.407, *SE =* 0.013) condition (Mean Difference = 0.032, *SE* = 0.007, p < 0.001) (Figure 4).
