# Supplementary material for: Motor Deficits in Schizophrenia Quantified by Nonlinear Analysis of Postural Sway
Source: PLoS One. 2012 Aug 1;7(8):e41808. doi: 10.1371/journal.pone.0041808 (PMC3411581; doi:10.1371/journal.pone.0041808)
Supplement: Supporting Information S5 — DFA-AP eyes X group interaction (singly versus dually diagnosed schizophrenia participants) post-hoc pair-wise comparisons. (DOC) [file pone.0041808.s005.doc]

**Supporting Information S5**

Motor Deficits in Schizophrenia Quantified by Nonlinear Analysis of Postural Sway

Jerillyn S. Kent,^1^ S. Lee Hong,^2^ Amanda R. Bolbecker,^1,3^ Mallory J. Klaunig,^4^ Jennifer K. Forsyth,^5^ Brian F. O’Donnell,^1,3,6^ & William P. Hetrick*^1,3,6^

1. Department of Psychological and Brain Sciences, Indiana University, Bloomington, Indiana, United States of America
2. Department of Biomedical Sciences, Ohio University, Athens, Ohio, United States of America
3. Department of Psychiatry, Indiana University School of Medicine, Indianapolis, Indiana, United States of America
4. Department of Cognitive Neuroscience, Ludwig Maximilian University of Munich, Munich, Germany
5. Department of Psychology, University of California Los Angeles, Los Angeles, California, United States of America
6. Larue D. Carter Memorial Hospital, Indianapolis, Indiana, United States of America

*corresponding author: whetrick@indiana.edu (email); 1-812-855-2620 (phone); 1-812-855-2012 (fax)

*Supporting Information S5: DFA-AP eyes X group interaction (singly versus dually diagnosed schizophrenia participants) post-hoc pair-wise comparisons*

Four post-hoc pair-wise comparisons were conducted comparing the eyes open versus eyes closed conditions within and between groups (α-level adjusted to p < 0.0125). Dually diagnosed individuals had greater DFA-AP values (decreased complexity) in the eyes open (*M =* 1.431, *SE =* 0.025) versus eyes closed (*M =* 1.397, *SE =* 0.028) conditions (Mean Difference = 0.034, *SE =* 0.012, p = 0.008).
